# For Whom Does It Work? Moderators of Outcome on the Effect of a Transdiagnostic Internet-Based Maintenance Treatment After Inpatient Psychotherapy: Randomized Controlled Trial

**DOI:** 10.2196/jmir.2511

**Published:** 2013-10-10

**Authors:** David Daniel Ebert, Mario Gollwitzer, Heleen Riper, Pim Cuijpers, Harald Baumeister, Matthias Berking

**Affiliations:** ^1^Innovation IncubatorDivision Health-Training.OnlineLeuphana University LueneburgLüneburgGermany; ^2^Division of Clinical Psychology and PsychotherapyDepartment of PsychologyPhilipps University of MarburgMarburgGermany; ^3^Division of Methodology and Social PsychologyDepartment of PsychologyPhilipps University of MarburgMarburgGermany; ^4^Department of Clinical Psychology & EMGO InstituteVU AmsterdamAmsterdamNetherlands; ^5^Department of Medical Psychology and Medical SociologyFaculty of MedicineUniversity of FreiburgFreiburgGermany; ^6^Department of Rehabilitation Psychology and PsychotherapyInstitute of PsychologyUniversity of FreiburgFreiburgGermany

**Keywords:** maintenance treatment, continuation treatment, Internet-based intervention, transdiagnostic treatment, mental disorders/inpatient psychotherapy, guided self-help, randomized controlled trial, relapse prevention, predictors, moderators

## Abstract

**Background:**

Recent studies provide evidence for the effectiveness of Internet-based maintenance treatments for mental disorders. However, it is still unclear which participants might or might not profit from this particular kind of treatment delivery.

**Objective:**

The study aimed to identify moderators of treatment outcome in a transdiagnostic Internet-based maintenance treatment (TIMT) offered to patients after inpatient psychotherapy for mental disorders in routine care.

**Methods:**

Using data from a randomized controlled trial (N=400) designed to test the effectiveness of TIMT, we performed secondary analyses to identify factors moderating the effects of TIMT (intervention) when compared with those of a treatment-as-usual control condition. TIMT involved an online self-management module, asynchronous patient–therapist communication, a peer support group, and online-based progress monitoring. Participants in the control condition had unstructured access to outpatient psychotherapy, standardized outpatient face-to-face continuation treatment, and psychotropic management. Self-reports of psychopathological symptoms and potential moderators were assessed at the start of inpatient treatment (T1), at discharge from inpatient treatment/start of TIMT (T2), and at 3-month (T3) and 12-month follow-up (T4).

**Results:**

Education level, positive outcome expectations, and diagnoses significantly moderated intervention versus control differences regarding changes in outcomes between T2 and T3. Only education level moderated change differences between T2 and T4. The effectiveness of the intervention (vs control) was more pronounced among participants with a low (vs high) education level (T2-T3: B=–0.32, SE 0.16, *P*=.049; T2-T4: B=–0.42, SE 0.21, *P*=.049), participants with high (vs low) positive outcome expectations (T2-T3: B=–0.12, SE 0.05, *P*=.02) and participants with anxiety disorder (vs mood disorder) (T2-T3: B=–0.43, SE 0.21, *P*=.04). Simple slope analyses revealed that despite some subgroups benefiting less from the intervention than others, all subgroups still benefited significantly.

**Conclusions:**

This transdiagnostic Internet-based maintenance treatment might be suitable for a wide range of participants differing in various clinical, motivational, and demographic characteristics. The treatment is especially effective for participants with low education levels. These findings may generalize to other Internet-based maintenance treatments.

**Trial Registration:**

International Standard Randomized Controlled Trial Number (ISRCTN): 28632626; http://www.controlled-trials.com/isrctn/pf/28632626 (Archived by WebCite at http://www.webcitation.org/6IqZjTLrx).

## Introduction

Despite strong evidence for the efficacy of psychotherapy for common mental health disorders [1,2], long-term outcome of psychotherapeutic interventions are still a major concern [3-6]. Psychological treatments following acute phase psychotherapy that aim to maintain achieved changes (ie, maintenance phase treatments) have been shown to enhance outcome sustainability (eg, major depressive disorder [7,8], obsessive compulsive disorder [9], and personality disorders [10,11]). However, such interventions are difficult to disseminate owing to high intervention costs and limited clinician availability.

The use of the Internet to provide guided self-help maintenance phase treatments may help to overcome this unmet maintenance need. Internet-based guided self-help strategies for the maintenance phase of psychotherapies have several advantages over face-to-face maintenance approaches. These include (1) greater potential for the integration of acquired skills in daily life because of an emphasis on the patient’s active role in (guided) self-help treatment [12], (2) elimination of waiting periods between acute and maintenance treatment, (3) elimination of travel time and costs for both patients and clinicians, (4) access to the programs on a 24/7 basis, and (5) lower costs.

Several studies have shown promising results with delivering maintenance phase treatments over the Internet [13-20]. For example, our group developed a form of Internet-based continuation phase psychotherapy, a transdiagnostic Internet-based maintenance treatment (TIMT) following inpatient psychotherapy [15,20]. TIMT was designed to increase long-term outcomes of inpatients treated in a routine care setting for common mental health disorders, such as major depressive, anxiety, posttraumatic stress, obsessive compulsive, eating, or somatoform disorders. Recently, TIMT was evaluated in a pragmatic randomized controlled trial (RCT), comparing TIMT in addition to treatment as usual (TAU) to TAU only (N=400). In this study, participants in the TIMT plus TAU condition showed a better maintenance of inpatient treatment effects (ie, differences in change of psychopathological symptom severity) from inpatient discharge to 3-month follow-up (between-group effect size: *d*=0.38, *P*<.001) and 12-month follow-up (between-group effect size: *d*=0.55, *P*<.001) than TAU-only controls [15].

Although there is evidence for the general effectiveness of Internet-based maintenance phase treatments, little is known about which patients might or might not benefit from this particular kind of treatment delivery. Investigating the moderating effects of patient characteristics on Internet-based maintenance phase treatment effectiveness is crucial for identifying appropriate populations and for customizing interventions to the specific needs of patient subgroups. More knowledge regarding who is likely or unlikely to profit from these interventions should also help in identifying relevant mechanisms of change as well as allocating health care resources on an evidence-based level [21].

Only a few studies to date have investigated moderators of Internet-based intervention outcomes for mental health problems. In 1 of these studies, Warmerdam and colleagues [22] explored moderators of Internet-based cognitive behavioral therapy (CBT) and Internet-based problem-solving therapy for depressive symptoms. None of the variables investigated in this study (demographic variables, illness severity, dysfunctional attitudes, and problem-solving skills) moderated the differential effectiveness of the 2 treatments. In a study comparing the effects of Internet-based CBT to group-based face-to-face CBT, Spek et al [23] found that participants high in altruism performed better in group CBT than in Internet-based CBT (no significant findings for age, gender, education, neuroticism, extraversion, agreeableness, openness and conscientiousness, pretreatment severity, previous episodes of depression, and marital status). When comparing responses to online CBT for depression compared to a waitlist control group Button et al [24] found that higher pretreatment severity of depressive symptoms were associated with a greater benefit of treatment. In another study, de Graaf and colleagues [25] explored pretreatment and short-term improvement variables as moderators of unsupported Internet-based CBT outcomes, usual primary care (TAU), and CBT combined with TAU for depression. They found that patients with higher levels of extreme positive responding to questionnaires had a better outcome in Internet-based CBT compared to TAU, whereas those with parental psychiatric history or with a major depressive disorder diagnosis had a better outcome in Internet-based CBT plus TAU compared to TAU.

The aim of the present study was to identify moderating factors on the effects of TIMT after inpatient psychotherapy. Using data from a pragmatic RCT on the effectiveness of TIMT (ISRCTN:28632626) [15], we conducted secondary analyses to identify demographic, clinical, and motivational variables that moderate the effects of TIMT on change in psychopathological symptom severity.

Given the current lack of data on moderators of Internet-based continuation phase treatment effects, we used an exploratory approach including a wide range of potential pretreatment moderators [21]. Our choice of moderators was based on (1) results of previous studies investigating moderators of face-to-face continuation treatment outcomes [26,27], (2) results of previous predictors/moderators in Internet-based intervention outcome studies [22,23,25,28,29], (3) predictors of relapse/long-term outcome studies [5,27,30-36], and (4) theoretical assumptions attributed to intervention characteristics. The final list of potential moderators investigated in the present study included (1) demographics, such as age, gender, education level, and computer/Internet literacy; (2) clinical characteristics, such as diagnoses, remission status, age of first onset, comorbid personality disorder, and reliable change during inpatient treatment, and (c) motivational variables, such as self-efficacy and positive outcome expectations.

The primary research questions of this study were:

1. Do any of the pretreatment factors included in this study moderate the effectiveness of TIMT compared with TAU?
2. If moderating effects are found, do participants characterized by disadvantageous scores on identified moderators still benefit from TIMT?

## Methods

### Study Design

We performed secondary analyses using data from a pragmatic RCT comparing TIMT in addition to TAU following inpatient psychotherapy to TAU only (N=400) [15]. The RCT was conducted in a German clinic providing routine mental health care. Study outcomes were assessed by using self-report measures that were completed at inpatient admission (T1), end of inpatient treatment/beginning of TIMT (T2), 3 months after discharge/end of TIMT (T3), and 12 months after inpatient treatment completion (T4). The study was powered to find a small to moderate effect size in the main effect analyses, which was considered to be the smallest relevant difference to health care decision makers in this context. All procedures were approved by the university and the hospital institutional review boards. Design and results of the effectiveness trial are described in detail in a previously published study [15].

### Participants and Procedures

We recruited potential participants from 2189 patients treated for a variety of mental disorders between July 2008 and October 2009 in the study hospital. Patients were eligible for the study if they (1) were age 18 years or older, (2) met criteria for a mental disorder according to the *International Classification of Diseases, Tenth Revision* (*ICD-10*) [37], (3) spoke German sufficiently, (4) had basic reading and writing skills, and (5) had access to a computer with an Internet connection. Exclusion criteria were (1) a psychotic diagnosis, (2) acute alcohol or substance dependence, and (3) a significant risk for suicide.

**Figure 1:**
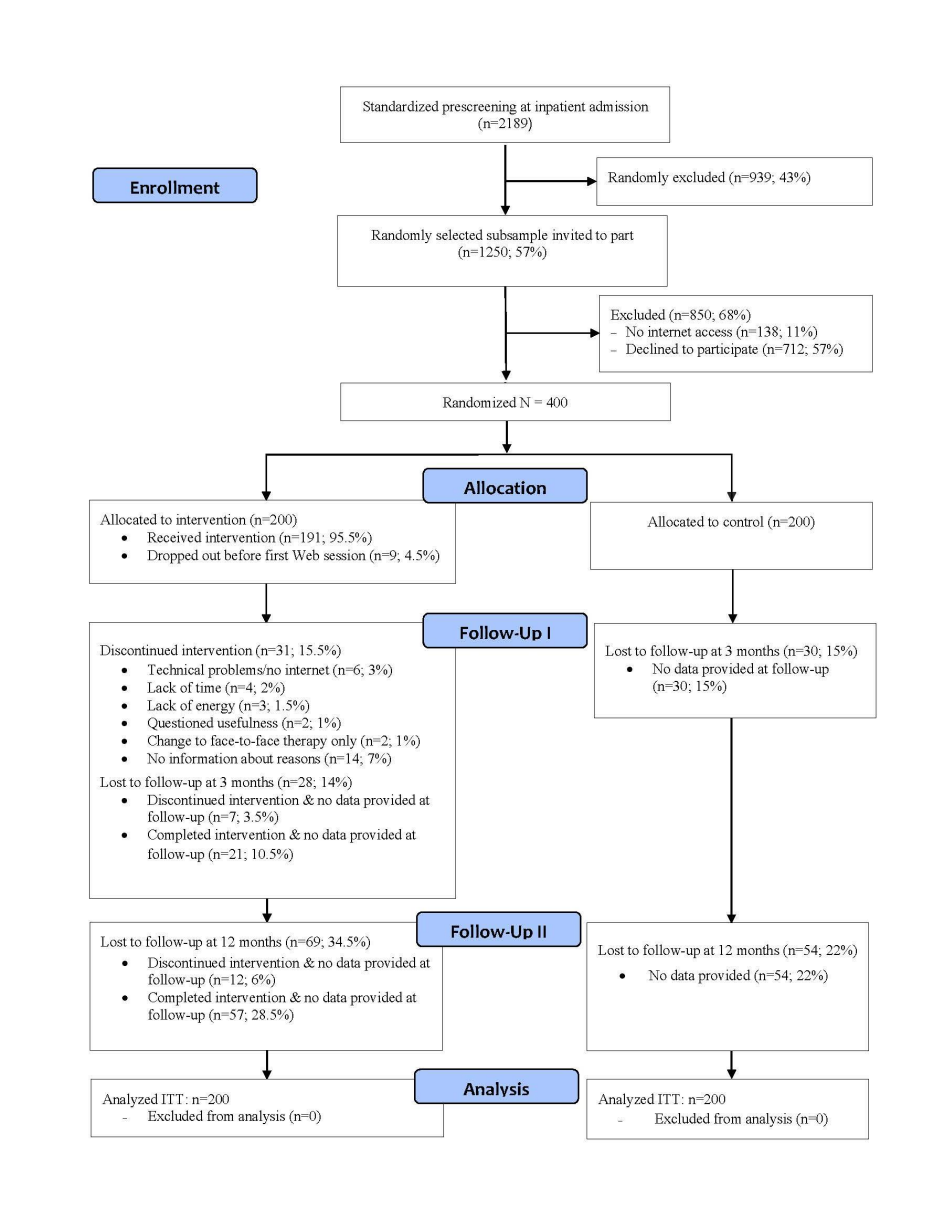
Participant flow and study dropouts at each stage of the study.

Participants who gave full written informed consent were randomly assigned to receive TAU only (control) or TAU plus TIMT (intervention). In total, 58 of 400 (14.5%) participants did not complete the T3 assessment and 113 (28.5%) did not complete the T4 assessment. Participants who did not provide data at 1 of the follow-ups did not differ from participants without missing data on baseline psychopathological symptom severity scores or any other clinical characteristics (all *P* values >.10), except for age (noncompleters on average 2.21 years younger than completers, *P*=.02). No significant interactions were found between missing pattern and outcome using pattern mixture analyses [38]. Thus, missing data appear not to bias the results. Figure 1 summarizes participant enrollment and flow throughout the study [15].

### Interventions

#### Inpatient Treatment

Inpatient treatment was based on CBT [39]. Participants received 1 session of individual therapy (50 minutes) and an average of 6 sessions of group therapy (90 minutes) per week. Interventions were supplemented with sports therapy and physiotherapy, as well as medical treatment (including pharmacotherapy) when necessary. Treatment was delivered by 6 experienced therapists and 14 therapists in training. Duration of treatment ranged between 22 and 98 days (mean 46.30, SD 8.17).

#### Treatment as Usual Condition

Following inpatient treatment, all participants had unstructured access to outpatient psychotherapy and standardized outpatient group-based, face-to-face, maintenance treatment [40] as typically provided by the referring agencies. In addition, there was no restriction on the use of medication during the study period.

#### Treatment as Usual Plus Transdiagnostic Internet-Based Maintenance Treatment Condition

In addition to TAU, the intervention group had TIMT for 12 weeks. The main focus of TIMT is to support patients in the sustained utilization of skills acquired during treatment. For this purpose, TIMT works to help participants identify activities that they have found helpful and systematically integrate these into their daily life routines. Because TIMT aims to enhance whatever strategy patients experienced as helpful, it can be used to maintain treatment outcome regardless of which psychopathology the patient is suffering from and regardless of the kind of treatment the patient received before. TIMT consists of 5 core components. The first component is the generation of a personal development plan. This process is conducted during the last 10 days of inpatient treatment in which TIMT participants complete 3 sessions of blended (face-to-face and online) standardized goal-setting and action planning instead of inpatient TAU. Participants develop a detailed plan including (1) highly relevant personal goals they want to achieve during the intervention phase, and (2) implementation intentions [41], including details on how and when they will achieve these goals. The second and central component of TIMT is the completion of a structured Web diary in which participants evaluate the realization of their personal goals weekly and set specific goals for the next week. The third component of TIMT is an online peer support group. Subgroups consisting of 3 to 6 participants are asked to give asynchronous online feedback to one another on their Web diaries. The fourth component of TIMT is coach support, involving weekly asynchronous written online feedback from a therapist regarding a participants’ Web diary. Coaches differed in their level of formal training, ranging from master’s level psychology students (n=1) and psychotherapists-in-training (n=1) to experienced CBT-trained psychotherapists with more than 10 years of professional experience (n=3). Coaches were supervised once a week by a licensed senior therapist, as is usual in the study hospital. Coaches were advised not to spend more than 30 minutes per week on support per patient. Total duration of support rendered was 231 minutes on average per patient (range: 10-490, SD 128). Finally, TIMT included weekly online monitoring of psychopathological symptoms.

#### Treatment Received

The intervention and control group did not differ in types of treatment received except for frequency of sedatives taken. Participants in the intervention group were less likely to take sedatives than controls (*P*<.001) [15].

### Measures

#### Moderators

In total, we included 11 pretreatment participant characteristics: age, sex, education, main diagnosis, comorbid personality disorder, remission status at the end of inpatient treatment, reliable change in the primary outcome during inpatient treatment, years since first disorder onset, Internet/computer literacy, positive outcome expectations, and health-related self-efficacy.

Information on sex, age, and education were extracted from the inpatient clinic patient files. All self-report data were assessed using an online-based assessment tool. Diagnoses and year of first disorder onsets were assessed during the intake interview. All interviewers were experienced psychotherapists who were either psychologists or physicians with a master’s degree or higher, trained extensively in administering the structured clinical interviews of the German version of the *Diagnostic and Statistical Manual of Mental Disorders* (Fourth Edition) (*DSM-IV*) [42]. Participants were classified as being remitted at inpatient discharge (yes/no) when individual scores in psychopathological symptom severity (primary outcome measure as described subsequently) exceeded a raw score value of 0.685 [43]). Reliable change in symptom severity (yes/no) was determined according to the widely used reliable change index of Jacobson and Truax [44]. Individual reliable change scores less than -1.96 were considered to reflect reliable (positive) change; scores equal to or greater than -1.96 reflected no reliable change. Participants were classified as Internet/computer illiterate if they checked the not at all response category for the item “I am used to sending and receiving emails” (1=not at all; 4=completely true). All other participants were classified as Internet/computer literate. Participants were coded as having a low education level if they reported 9 years of school education, as a medium education level if they reported 10 years of school education and a corresponding degree, or as a high education level if they reported a minimum of 13 years of school/college education and a corresponding degree.

Positive outcome expectations were assessed by using the respective subscale of the Patient Questionnaire on Therapy Expectation and Evaluation (PATHEV) [45]. This scale consists of 4 items measuring participants’ expectations regarding the effectiveness of their inpatient treatment (eg, “I think that finally my problems will be solved”). Response scales ranged from 0 (=do not agree) to 4 (=agree completely). Higher scores reflect higher positive outcome expectations. Construct validity of the scale was demonstrated in several studies [45]. In the present study, internal consistency (Cronbach alpha) was .77.

Health-related self-efficacy was assessed by using the self-efficacy subscale of the 49-item short form of the Hamburg Modules for the Assessment of Psychosocial Health (HEALTH-49) questionnaire [43].This scale includes 5 items measuring expected persistence and success in several domains (eg, “Despite my discomfort, I achieve the personal goals that I set for myself” score inverted for scale calculation; 0=not true, 4=very true). Higher scores denote lower self-efficacy. Internal consistency (Cronbach alpha) was .86 in the present study.

#### Dependent Variable

The primary outcome from the effectiveness trial was change in general psychopathological symptom severity (symptom severity) from discharge (T2) to 3- and 12-month follow-ups (T3, T4). Symptom severity was assessed by using the HEALTH-49, a widely used measure of symptom severity in Germany [43]. The HEALTH-49 general psychopathological symptom severity scale consists of 18 items related to somatoform complaints (7 items), depressiveness (6 items), and phobic anxiety (5 items). Participants were asked to rate the severity to which they had suffered from the presented symptoms in the previous 2 weeks (0=not at all; 4=very much). Reliability and construct validity have been established in several studies based on large clinical and nonclinical samples (1548 psychotherapy inpatients, 5630 primary care patients, see [43]). In the present study, internal consistency (Cronbach alpha coefficients) at baseline was .87 for the overall general psychopathological symptom severity score, 0.90 for depressive symptoms, 0.86 for somatoform complaints, and 0.86 for phobic anxiety.

### Statistical Analyses

Group differences regarding baseline characteristics were compared via chi-square tests for categorical variables and *t* tests for continuous variables. Interactions between pretreatment participant characteristics (moderators) and interindividual differences in intraindividual changes across measurement occasions were modeled and tested via multilevel mixed-effect models. Change in symptom severity over time was dummy coded and treated as a fixed level-1 (ie, within-subjects) effect (dummy 1: T1-T2, dummy 2: T2-T3, dummy 3: T2-T4). Treatment conditions (0=control condition, 1=intervention condition) was treated as a fixed level-2 (ie, between-subjects) effect. More important for the present purpose, interactions between moderator and treatment condition, all cross-level interaction effects (condition × T1-T2, T2-T3, T2-T4; moderator × T1-T2, T2-T3, T2-T4), and 3-way interaction effects (moderator × condition × T1-T2, T2-T3, T2-T4) were also included in the models. A 3-way interaction effect of moderator × condition × T2-T3 or moderator × condition × T2-T4 would indicate that the magnitude of the intervention effect varies as a function of the moderator. The model imposed no restrictions on the covariance matrix for measurement occasions. Thus, no model assumptions were tested. We standardized continuous predictors so that regression coefficients were estimated for participants with average scores on the putative moderator.

To increase interpretability and allow for testing nonlinear effects, categorical variables with more than 2 categories (ie, diagnosis, years since first disorder onset, education) were recoded into a maximum of 3 meaningful categories. Because of low prevalence rates, we excluded diagnoses other than depression, anxiety disorders, and adjustment disorders. All continuous moderators (ie, age, self-efficacy, positive outcome expectations) were standardized so that regression coefficients refer to participants with average scores on each moderator.

Aiming at an intention-to-treat (ITT) design, we included all participants randomly assigned to conditions. We employed a full information maximum likelihood (FIML) estimation, which allows for all available data to be included without replacement or imputation of missing values. The FIML estimation for mixed models is especially robust with respect to missing data [46].

Additionally, we conducted follow-up simple slope analyses for each significant 3-way interaction effect [47] to probe the relevant lower-order effects. In this method, the slope and the significance of the intervention main effect is evaluated for conditional values of the moderator. For significant 3-way interactions of continuous moderators, simple slopes were calculated for the mean and one standard deviation above and below the mean [48].

Effect sizes for each significant moderator were calculated based on comparing the effect of control versus intervention groups on symptom severity scores, with participants grouped by the significant moderator variable. Cohen’s *d* scores [49] were calculated by standardizing the differences between baseline and follow-up by the pooled standard deviation of baseline scores.

To verify whether the results of the ITT analyses would be sustained among the intervention completers sample only, we subsequently repeated all mixed-effects models with participants who stayed within key treatment parameters (completed at least 6 of 12 Web diary entries or more than 25 posts, n=177).

To clarify the generalizability of our findings, we assessed all potential moderators also from patients who were treated during the recruitment period in the study center, but did not participate in the trial (not invited, declined to participate, not fulfilling inclusion criteria) but gave informed consent to use their data for research purposes (n=1789). Study participants and nonparticipants were compared using chi-square tests for categorical variables and *t* tests for continuous variables.

Finally, if a significant moderator effect contradicted our a priori expectations, we conducted post hoc simple slope analyses for the control and intervention groups separately to identify the reasons for the effect. All analyses were performed with SPSS 19 (IBM Corp, Armonk, NY, USA).

### Descriptive Data

Table 1 shows descriptive statistics for the dependent variable general psychopathological symptom severity. Table 2 shows descriptive data for all moderator variables. Table 1 and parts of Table 2 have been reported in previous studies [15]. Consistent with random assignment, no differences were found between intervention and control group on any of the pretreatment variables.

**Table 1:** Descriptives for primary trial main outcome, psychopathological symptom severity as measured by the general psychopathological symptom severity subscale of the HEALTH-49 questionnaire.

Assessment points	Time of Assessment	Intervention (n=200)		Control (n=200)	
		Mean	SD	Mean	SD
T1	Inpatient admission	1.50	0.69	1.49	0.71
T2	Inpatient discharge	0.83	0.64	0.83	0.66
T3	3-month follow-up	0.71	0.61	0.96	0.69
T4	12-month follow-up	0.78	0.69	1.12	0.84

**Table 2:** Descriptives for pretreatment moderator variables.

Variables		Intervention (n=200)	Control (n=200)	Nonparticipants^a^ (n=1789)
Age, mean (SD)		45.09 (8.88)	45.45 (9.80)	47.12 (9.45)
Sex (female), n (%)		147 (73.5)	151 (75.5)	1360 (76.0)
**Education, n (%)**				
	High	80 (40.0)	78 (39.0)	498 (27.8)
	Medium	93 (46.5)	91 (45.5)	779 (43.5)
	Low	26 (13.0)	31 (15.5)	509 (28.5)
Existing Internet literacy (%)		178 (89.0)	167 (83.5)	1132 (67.5)^b^
**Disorder, n (%)**				
	Mood disorder	108 (54.0)	113 (56.5)	918 (51.3)
	Anxiety	19 (9.5)	18 (9.0)	206 (11.5)
	Adjustment	53 (26.5)	38 (19.0)	405 (22.6)
	Other	20 (10.0)	31 (15.5)	260 (14.5)
Comorbid personality disorder, n (%)		20 (10.0)	22 (11.0)	175 (9.8)
**Years since first disorder onset (years) n (%)**				
	< 1	44 (22.0)	47 (23.5)	430 (24.2)
	1-5	55 (27.5)	44 (22.0)	444 (24.9)
	> 5	96 (48.0)	105 (52.5)	906 (50.9)
Reliable change during inpatient treatment, n (%)		100 (50.0)	90 (45.0)	1052 (58.8)
Remission at discharge, n (%)		94 (47.0)	93 (46.5)	787 (44.0)
Self-efficacy, mean (SD)		1.47 (0.83)	1.49 (0.87)	1.58 (0.90)
Positive outcome expectations, mean (SD)		3.86 (0.74)	3.92 (0.66)	3.72 (0.78)

^a^All differences between conditions were nonsignificant. If percentages do not reach 100, it is due to missing data.

^b^n=1676.

## Results

### Moderators of Treatment Outcome

#### Overview

The subsequent tables show the mixed-effect model results based on ITT for the interactions between pretreatment participant characteristics (moderators), intervention condition, and changes in symptom severity. Intercepts represent the estimated level of symptom severity at baseline (discharge, T2). The regression coefficient of the moderator represents differences in symptom severity between participants differing in 1 unit of the hypothesized moderator at baseline. The regression coefficient of T1-T2 represents the average difference in symptom severity between inpatient admission (T1) and inpatient discharge (T2) in the control group, the regression coefficient of T2-T3 represents the average difference in symptom severity between discharge (T2) and 3-month follow-up (T3) in the control group, and the regression coefficient of T2-T4 represents the average difference in symptom severity between discharge (T2) and 1-year follow-up (T4) in the control group. The regression coefficient of the condition represents differences in symptom severity between the intervention and the control condition at discharge (T2). The cross-level interactions condition × T1-T2, T2-T3, T2-T4 represent intervention versus control group differences in changes over time.

As expected, we found (1) a significant decrease in symptom severity between T1 and T2 in both conditions (T1-T2), (2) no interaction between T1-T2 and the intervention condition, (3) a significant condition × T2-T3 interaction effect showing that symptom severity remained low in the intervention group between T2 and T3 but increased in the control group, and (4) a significant T2-T4 × condition interaction effect showing that symptom severity remained low in the intervention group between T2 and T4 but increased in the control group (Table 4). The regression coefficients of the moderator × T1-T2, T2-T3, T2-T4 interaction effects represent moderator effects on changes in symptom severity across measurement occasions. Finally, the regression coefficient of condition × moderator × T1-T2, T2-T3, T2-T4 interaction effects represent moderator effects on intervention versus control condition differences on change scores over time.

#### Dichotomous Moderator Variables

Table 3 shows results for dichotomous moderator variables. The results revealed no moderator effects on intervention versus control group differences on changes in symptom severity over time (see Table 3, condition × moderator × T1-T2, T2-T3, T2-T4). Thus, none of the dichotomous moderators reliably altered the effectiveness of intervention versus control on symptom severity over time. The intervention was superior to control with regard to outcome sustainability, irrespective of sex, Internet literacy, reliable changes during inpatient treatment, comorbid personality disorder, or remission status at T2.

**Table 3:** Multilevel results of the interactions between pretreatment participant characteristics (dichotomic moderator variables), intervention condition, and change in psychopathological symptom severity (dummy coded) for the intention-to-treat sample (N=400) using full maximum likelihood estimation.

Interaction terms	Sex^a^			Internet literacy^b^			Reliable change^c^			Comorbid PD^d^			Remission status^e^		
	B	SE	*P*	B	SE	*P*	B	SE	*P*	B	SE	*P*	B	SE	*P*
Intercept^f^	0.94	0.09	<.001	0.91	0.11	<.001	0.98	0.06	<.001	0.78	0.05	<.001	0.30	0.04	<.001
Moderator	–0.16	0.11	.13	–0.11	0.12	.39	–0.34	0.09	<.001	0.42	0.15	.004	0.98	0.06	<.001
T1-T2 (dummy 1)^g^	0.60	0.08	<.001	0.69	0.10	<.001	0.25	0.03	<.001	0.66	0.04	<.001	0.84	0.06	<.001
T2-T3 (dummy 2)^h^	0.20	0.08	.008	0.19	0.09	.04	0.00	0.05	.99	0.16	0.04	<.001	0.36	0.05	<.001
T2-T4 (dummy 3)^i^	0.48	0.10	<.001	0.40	0.12	.001	0.12	0.06	.05	0.30	0.05	<.001	0.44	0.07	<.001
Condition^j^	–0.35	0.13	.006	–0.13	0.18	.48	0.05	0.09	.58	0.02	0.07	.78	0.01	0.06	.90
Condition×T1-T2	0.04	0.12	.750	0.07	0.16	.66	–0.06	0.05	.25	0.04	0.06	.53	–0.04	0.08	.61
Condition×T2-T3	–0.24	0.11	.03	–0.30	0.14	.04	–0.22	0.07	.002	–0.24	0.06	<.001	–0.30	0.07	<.001
Condition×T2-T4	–0.45	0.14	.001	–0.65	0.18	<.001	–0.41	0.10	<.001	–0.34	0.07	<.001	–0.30	0.10	.002
Moderator×T1-T2	0.09	0.10	.35	–0.03	0.11	.80	0.92	0.05	<.001	0.02	0.13	.87	–0.31	0.08	<.001
Moderator×T2-T3	–0.04	0.09	.61	–0.02	0.10	.83	0.36	0.07	<.001	0.04	0.12	.75	–0.37	0.07	<.001
Moderator×T2-T4	–0.22	0.11	.05	–0.11	0.13	.41	0.41	0.09	<.001	0.08	0.16	.61	–0.25	0.10	.01
Condition×moderator	0.46	0.15	.002	0.14	0.19	.47	–0.07	0.13	.57	–0.23	0.21	.28	–0.02	0.09	.83
Cond×mod×T1-T2^k^	–0.03	0.14	.84	–0.06	0.17	.72	0.05	0.07	.47	–0.25	0.19	.20	0.11	0.12	.36
Cond×mod×T2-T3^k^	–0.02	0.12	.89	0.06	0.15	.71	–0.08	0.10	.46	–0.12	0.18	.49	0.12	0.10	.22
Cond×mod×T2-T4^k^	0.12	0.16	.47	0.34	0.20	.09	0.06	0.14	.67	–0.22	0.23	.34	–0.11	0.14	.41

^a^Sex (0=female; 1=male).

^b^Existing Internet literacy (0=no; 1=yes).

^c^Reliable change: reliable change during inpatient treatment (0=no; 1=yes).

^d^Comorbid PD: comorbid personality disorder (0=no; 1=yes).

^e^Remission status: remission status at baseline (T2) (0=in remission; 1=not in remission).

^f^Intercept: general psychopathological symptom severity in control at baseline (T2).

^g^T1-T2: dummy-coded change in general psychopathological symptom severity from T1 to T2.

^h^T2-T3: dummy-coded change in general psychopathological symptom severity from T2 to T3.

^i^T2-T4: dummy-coded change in general psychopathological symptom severity from T2 to T4.

^j^Condition (0=control; 1=intervention).

^k^Cond × mod: condition × moderator.

#### Trichotomous Moderator Variables

**Figure 2:**
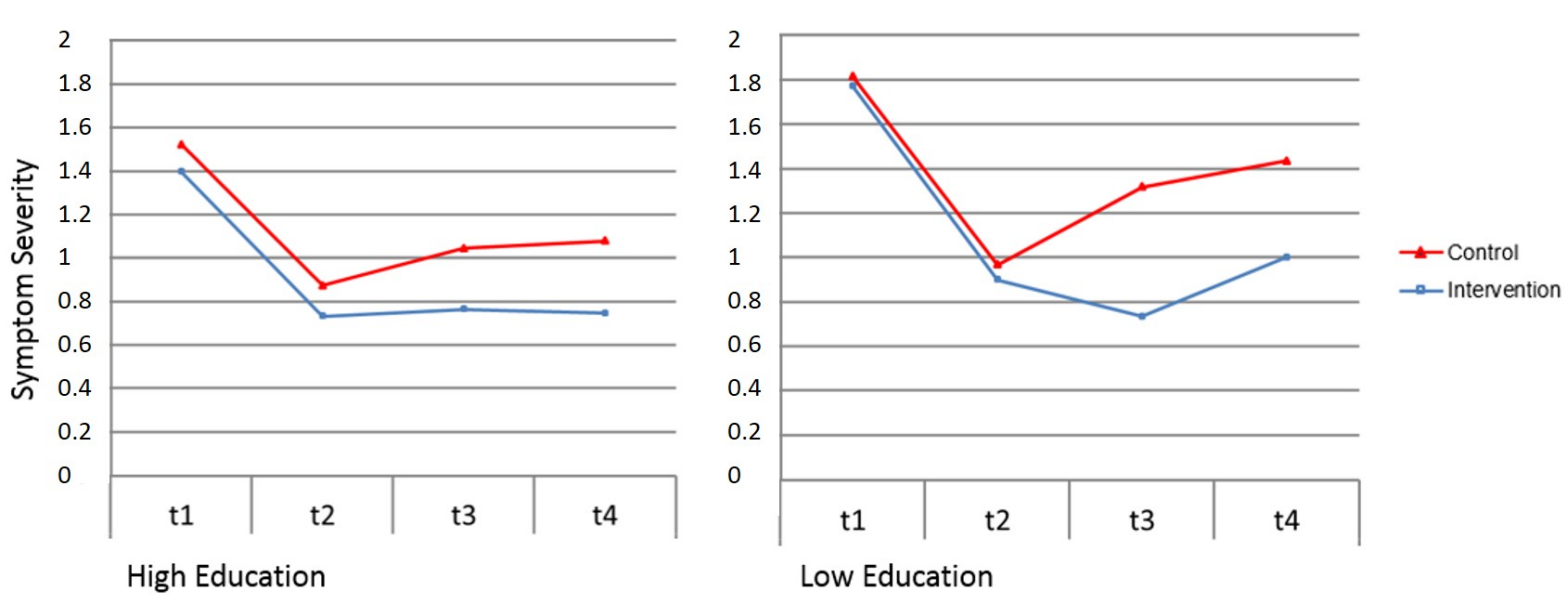
Estimated course of symptoms based on simple slope mixed-effect model analysis for significant moderators effect of education (0=high education, n=159; 1=low education, n=57) at inpatient admission (T1), inpatient discharge/begin transdiagnostic Internet-based maintenance treatment (T2), 3-month follow-up/end transdiagnostic Internet-based maintenance treatment (T3), and 12-month follow-up (T4).

Table 4 shows mixed-effects model results for the 3 trichotomous moderator variables education level, diagnoses, and years since first disorder onset. Three significant 3-way interaction effects were found. Education dummy 2 (low vs high education) interacted with condition × T2-T3, and with condition × T2-T4. These interactions indicate that a greater intervention effect was found among participants with low compared to high education level (see Figure 2). Participants low in education showed a larger intervention vs control condition difference on changes in symptom severity between discharge and 3-month follow-up and between discharge and 1-year follow-up. Post hoc analyses demonstrated that although simple slopes for the intervention main effects (condition × T2-T3, T2-T4) were lower among high-educated participants compared to low-educated participants, the intervention main effect was still significant (simple slope high-educated participants T2-T3: B=–0.17, SE 0.08, *P*=.04; T2-T4: B=–0.25, SE 0.11, *P*=.03; simple slope low-educated participants T2-T3: B=–0.49, SE 0.14, *P*<.001; T2-T4: B=–0.66, SE 0.18, *P*<.001).

**Figure 3:**
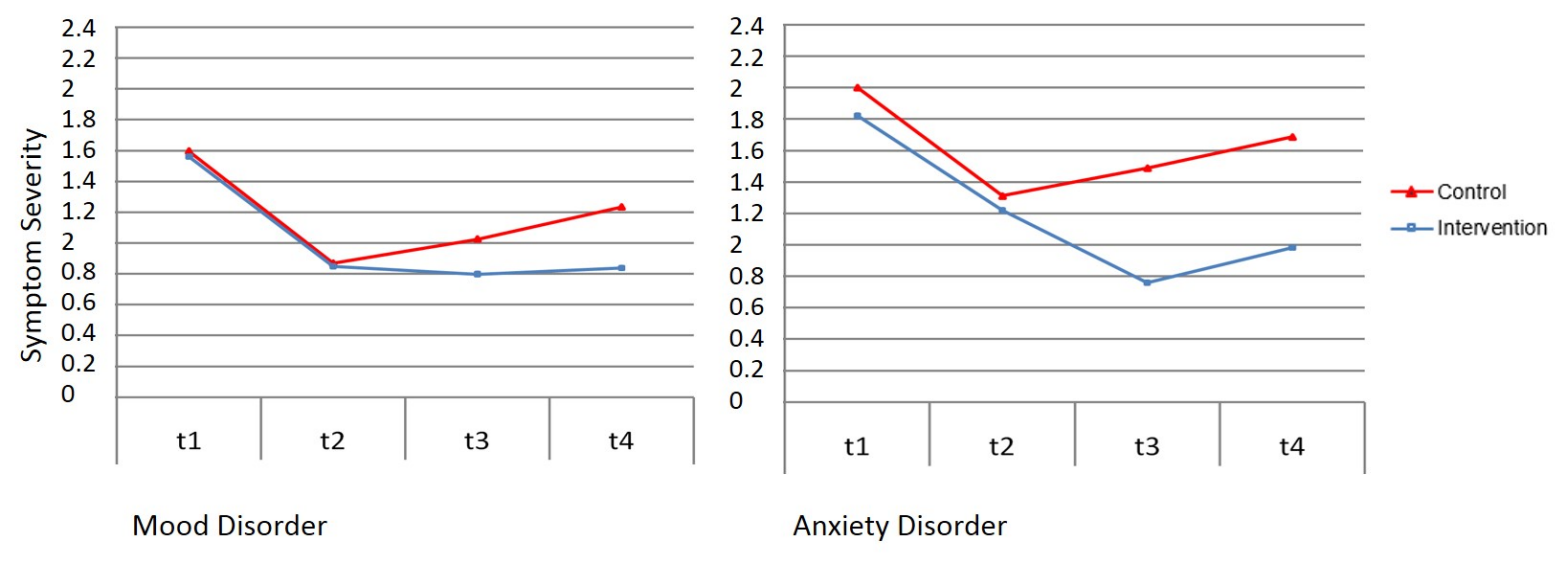
Estimated course of symptoms based on simple slope mixed-effect model analyses for significant moderator effect of diagnoses (0=mood disorder, n=221; 1=anxiety disorder, n=37) at inpatient admission (T1), inpatient discharge/begin transdiagnostic Internet-based maintenance treatment (T2), 3-month follow-up/end transdiagnostic Internet-based maintenance treatment (T3), and 12-month follow-up (T4).

Moreover, diagnoses dummy 1 (mood disorders vs anxiety disorders) interacted with condition × T2-T3. Participants diagnosed with an anxiety disorder showed a larger intervention versus control group difference on changes in symptom severity between discharge and 3-month follow-up than participants diagnosed with a mood disorder (see Figure 3). Post hoc analyses demonstrate that although simple slopes for the intervention main effect (condition × T2-T3) were lower among participants with a mood disorder compared to participants with an anxiety disorder, the intervention main effect was significant in both groups (simple slope mood disorder T2-T3: B=–0.21, SE 0.07, *P*=.004; simple slope anxiety disorder T2-T3: B=–0.64, SE 0.02, *P*<.001). Diagnoses dummy 1 did not moderate the association between treatment and change in symptom severity from discharge to 1-year follow-up.

Years since disorder onset did not moderate the effect of treatment on any intervention versus control group differences on change scores. Thus, transdiagnostic Internet-based maintenance treatment is effective irrespective of years since first disorder onset.

**Table 4:** Multilevel results for interactions between pretreatment participant characteristics (trichotomous moderator variables), intervention condition, and change in psychopathological symptom severity (dummy coded) for the intention-to-treat sample (N=400) using full maximum likelihood estimation.

Interaction terms	Education level^a^			Diagnoses^b^			Years since onset^c^		
	B	SE	*P*	B	SE	*P*	B	SE	*P*
Intercept^d^	0.88	0.07	<.001	0.87	0.06	<.001	0.88	0.09	<.001
Moderator dummy 1	–0.16	0.10	.11	0.44	0.16	.005	–0.04	0.11	.687
Moderator dummy 2	0.08	0.14	.55	–0.34	0.12	.004	–0.13	0.13	.313
T1-T2^e^	0.63	0.07	<.001	0.73	0.06	<.001	0.64	0.09	<.001
T2-T3^f^	0.17	0.06	.005	0.16	0.05	.002	0.10	0.08	.18
T2-T4^g^	0.22	0.08	.004	0.36	0.06	<.001	0.31	0.10	.002
Condition^h^	–0.14	0.10	.17	–0.02	0.08	.82	–0.28	0.13	.02
Condition×T1-T2	0.03	0.09	.76	–0.02	0.08	.85	0.09	0.12	.43
Condition×T2-T3	–0.17	0.08	.04	–0.21	0.07	.004	–0.20	0.10	.06
Condition×T2-T4	–0.25	0.11	.03	–0.38	0.09	<.001	–0.33	0.14	.02
Moderator dummy 1×T1-T2	0.03	0.09	.72	–0.04	0.15	.80	–0.04	0.11	.70
Moderator dummy 1×T2-T3	–0.04	0.08	.64	0.02	0.15	.89	0.04	0.09	.68
Moderator dummy 1×T2-T4	0.07	0.11	.48	0.01	0.19	.96	–0.03	0.12	.80
Moderator dummy 2×T1-T2	0.17	0.13	.17	–0.09	0.11	.40	0.20	0.12	.10
Moderator dummy 2×T2-T3	0.10	0.11	.35	0.03	0.10	.75	0.14	0.11	.19
Moderator dummy 2×T2-T4	0.35	0.14	.02	–0.19	0.13	.14	0.02	0.14	.88
Cond×mod×dummy 1^i^	0.22	0.14	.11	–0.07	0.22	.74	0.36	0.15	.02
Cond×mod×dummy 2^i^	0.19	0.20	.34	0.08	0.16	.63	0.27	0.18	.13
Cond×mod×dummy 1×T1-T2^i^	–0.03	0.13	.82	–0.07	0.22	.75	–0.07	0.15	.63
Cond×mod×dummy 1×T2-T3^i^	–0.08	0.11	.50	–0.43	0.21	.04	–0.03	0.13	.82
Cond×mod×dummy 1×T2-T4^i^	–0.12	0.15	.42	–0.24	0.26	.37	0.01	0.17	.93
Cond×mod×dummy 2×T1-T2^i^	0.05	0.18	.77	0.10	0.15	.52	–0.14	0.17	.43
Cond×mod×dummy 2×T2-T3^i^	–0.32	0.16	.049	–0.03	0.14	.83	–0.09	0.15	.56
Cond×mod×dummy 2×T2-T4^i^	–0.42	0.21	.049	0.15	0.18	.41	0.03	0.19	.89

^a^Education level dummy 1 (0=high education level; 1=medium education level), education level dummy 2 (0=high education level; 1=low education level).

^b^Diagnoses dummy 1 (0=mood disorder; 1=anxiety disorder), diagnoses dummy 2 (0=mood disorder; 1=adjustment disorder).

^c^Years since onset: years since disorder onset dummy 1 (0=1-5 years; 1=>5 years), years since disorder onset dummy 2 (0=1-5 years; 1=<1 year).

^d^Intercept: general psychopathological symptom severity in control at baseline (T2).

^e^T1-T2: dummy-coded change in general psychopathological symptom severity from T1 to T2.

^f^T2-T3: dummy-coded change in general psychopathological symptom severity from T2 to T3.

^g^T2-T4: dummy-coded change in general psychopathological symptom severity from T2 to T4.

^h^Condition (0=control; 1=intervention).

^i^Cond × mod × dummy: condition × moderator × dummy.

#### Continuous Moderator Variables

**Figure 4:**
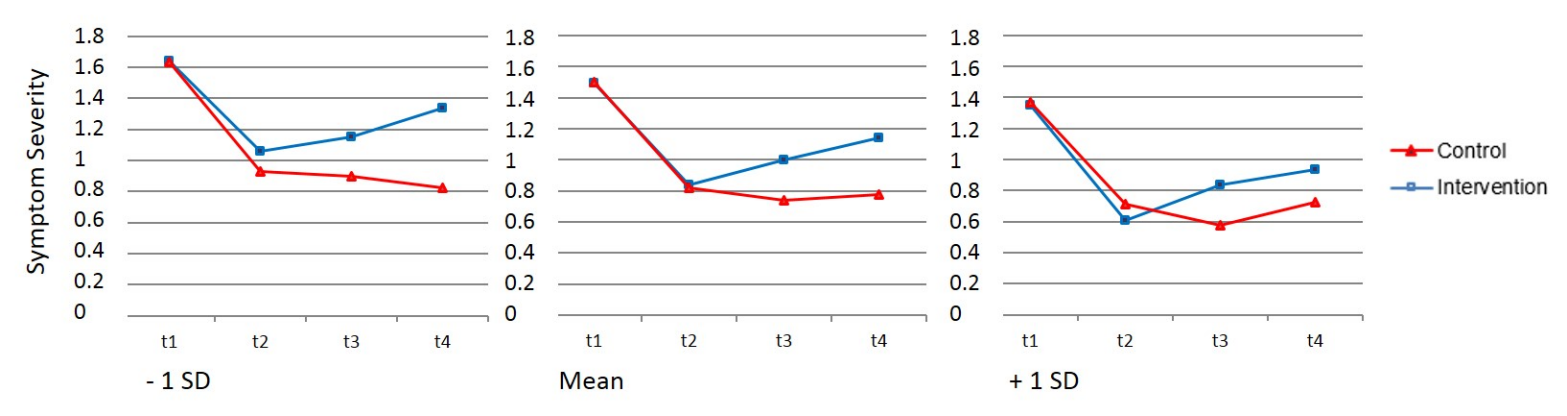
Estimated course of symptoms based on simple slope mixed-effect model analyses for significant moderator positive outcome expectations (mean vs mean – 1 SD vs mean + 1 SD) at inpatient admission (T1), inpatient discharge/begin transdiagnostic Internet-based maintenance treatment (T2), 3-month follow-up/end transdiagnostic Internet-based maintenance treatment (T3), and 12-month follow-up (T4).

Table 5 shows mixed-effect model results for the continuous moderator variables age, self-efficacy, and positive outcome expectations. One significant 3-way interaction was found. Positive outcome expectations interacted with condition × T2-T3. This interaction indicates that more positive outcome expectations were associated with stronger intervention effects between discharge and 3-month follow-up (see Figure 4). Follow-up analyses revealed that although simple slopes for the intervention main effect (condition × T2-T3) were lower among participants with a moderate (mean) positive outcome expectation than for participants with a high (mean + 1 SD) positive outcome expectations, the intervention effect was still significant (simple slope moderate positive outcome expectations T2-T3: B=–0.25, SE 0.05, *P*<.001; simple slope high positive outcome expectations T2-T3: B=–0.36, SE 0.07, *P*<.001). For participants with a low positive outcome expectations (mean – 1 SD), the simple slope for the intervention main effect (condition × T2-T3) was lower and no longer significant (simple slope low positive outcome expectations T2-T3: B=–0.13, SE 0.07, *P*=.09). Only 14.4% of participants (57/400) expressed low positive outcome expectations. Therefore, the drop to nonsignificance was likely because of low power. Moreover, simple slope analyses for this participant group showed that the intervention main effect on change in symptom severity from discharge to 1-year follow-up was significant (simple slope low positive outcome expectations T2-T4: B=–0.38, SE 0.10, *P*<.001). Although short-term effects were not significant, participants with low positive outcome expectations benefited in the long term from the intervention. There was no interaction between positive outcome expectations and change in symptom severity from discharge to 1-year follow-up and no interaction effect including the other continuous variables age and self-efficacy. Thus, TIMT seems to be effective irrespective of age and self-efficacy.

**Table 5:** Multilevel results for interactions between pretreatment participant characteristics (continuous moderator variables), intervention condition, and change in psychopathological symptom severity (dummy coded) for intention-to-treat sample (N=400) using full maximum likelihood estimation.

Interaction terms	Age^a^			Self efficacy^a^			Positive outcome expectations^a^			
	B	SE	*P*	B	SE	*P*	B	SE	*P*	
Intercept^b^	0.83	0.05	.<001	0.82	0.04	<.001	0.84	0.04	<.001	
Moderator^c^	–0.12	0.04	.004	0.44	0.03	<.001	–0.23	0.05	<.001	
T1-T2^d^	0.67	0.04	<.001	0.67	0.04	<.001	0.66	0.04	<.001	
T2-T3^e^	0.17	0.04	<.001	0.17	0.04	<.001	0.16	0.04	<.001	
T2-T4^f^	0.31	0.05	<.001	0.31	0.05	<.001	0.30	0.05	<.001	
Condition^g^	–0.01	0.06	.85	0.01	0.05	.89	–0.01	0.06	.84	
Condition×T1-T2	0.02	0.06	.79	0.01	0.06	.88	0.02	0.06	.75	
Condition×T2-T3	–0.24	0.05	<.001	–0.25	0.05	<.001	–0.25	0.05	<.001	
Condition×T2-T4	–0.35	0.07	<.001	–0.36	0.07	<.001	–0.35	0.07	<.001	
Moderator×T1-T2	0.01	0.04	.79	–0.18	0.04	<.001	0.08	0.04	.08	
Moderator×T2-T3	0.07	0.04	.07	–0.16	0.04	.002	0.07	0.04	.08	
Moderator×T2-T4	0.05	0.05	.26	–0.05	0.05	.26	0.03	0.05	.62	
Condition×moderator	0.03	0.06	.60	–0.04	0.05	.42	0.12	0.06	.06	
Condition×moderator×T1-T2	0.03	0.06	.60	0.05	0.06	.39	–0.10	0.06	.09	
Condition×moderator×T2-T3	0.04	0.05	.50	0.07	0.05	.22	–0.12	0.05	.02	
Condition×moderator×T2-T4	0.00	0.07	.98	–0.10	0.07	.15	0.03	0.07	.65	

^a^All continuous variables standardized.

^b^Intercept: general psychopathological symptom severity in control at baseline (T2).

^c^Moderators (0=mean; 1=mean + 1 SD).

^d^T1-T2: dummy-coded change in general psychopathological symptom severity from T1 to T2.

^e^T2-T3: dummy-coded change in general psychopathological symptom severity from T2 to T3.

^f^T2-T4: dummy-coded change in general psychopathological symptom severity from T2 to T4.

^g^Condition (0=control; 1=intervention).

#### Effect Sizes

Effect sizes (Cohen’s *d*) for each significant moderator were calculated based on comparing the effect of control versus intervention condition on symptom severity, with participants grouped by parameter values on each significant moderator variable. A mean effect size of *d*=0.22 was found for participants with high education and *d*=0.80 for participants with low education for control versus intervention group differences in change of psychopathological symptom severity from discharge to 3-month follow-up. For change from discharge to 1-year follow-up, a mean effect size of *d*=0.30 for high-educated participants and a mean effect size of *d*=0.57 for low-educated participants was found. With diagnoses as the moderator, control versus intervention group differences in change from discharge to 3-month follow-up were *d*=0.33 for participants with a mood disorder and *d*=1.02 for participants with an anxiety disorder. With positive outcome expectations as moderator, control versus intervention group differences in change from discharge to 3-month follow-up were *d*=0.58 for participants with high positive outcome expectations, *d*=0.39 for participants with mean positive outcome expectations, and *d*=0.20 for participants with low positive outcome expectations.

### Intervention Completers Sample

The results of the following intervention completers analyses closely paralleled those of the ITT analyses. Most of the significant 3-way interactions were also significant in the completers sample (B=–0.45 to –0.12, SE 0.05-0.21, *P*=.03-.046). Only the interaction of education dummy 2 with condition × T2-T4 was no longer significant at follow-up (B=–0.43, SE 0.22, *P*=0.05). None of the nonsignificant interactions in the ITT analyses was significant in the completers sample (B=–0.11 to 0.02, SE 0.07-0.20, *P*=.08-.97).

### Generalizability

As shown in Table 1 and partly reported in previous studies [15], study participants did not differ from nonparticipants (n=1789) regarding sex (χ^2^_1_=0.4, *P*=.52), years since first disorder onset (χ^2^_2_=0.1, *P*=.93), existing comorbid personality disorder (χ^2^_1_=0.5, *P*=.46), or remission status at the end of inpatient treatment (χ^2^_1_=1.0, *P*=.32) or initial psychopathological symptom severity at inpatient admission (study participant symptom severity T1: mean 1.49, SD 0.70; nonparticipant symptom severity T1: mean 1.52, SD 0.84, *t*_679.03_=–0.69, *P*=.54). Study participants were significantly younger than nonparticipants (with an average difference of 1.7 years, *t*_2135_=–3.54, *P*<.001), had higher self-efficacy (*t*_2182_=–2.11, *P*=.04, *d*=0.15) had a slightly higher education level (χ^2^_2_=40.81, *P*<.001, Kendall’s tau coefficient=0.11), had higher positive outcome expectations (*t*_625.6_=4.07, *P*<.001, *d*=0.27). Compared to nonparticipants, a greater percentage of participants had access to the Internet (χ^2^_1_=47.3, *P*<.001, phi coefficient=0.15) were Internet literate (χ^2^_1_=62.7, *P*<.001, phi coefficient=0.17), and relatively fewer showed reliable change during inpatient treatment (χ^2^_1_=5.3, *P*=.02, phi coefficient=0.05).

### Post Hoc Analyses

The moderator effect of education contradicted our a priori expectation of higher educated participants benefiting to a greater extent from the Internet-based intervention than lower educated participants. Thus, we conducted further post hoc simple slope analyses for the control group and the intervention group separately to identify possible explanations for this effect. For participants in the control group, we found no significant interaction between education and changes in symptom severity from discharge to 3-month follow-up (education dummy 2 × T2-T3 interaction, B=0.10, SE 0.11, *P*=.35), but we found a significant interaction between education and changes from discharge to 12-month follow-up (education dummy 2 × T2-T4, B=0.35, SE 0.14, *P*=.02). Less-educated participants had a greater risk for deterioration from discharge to 1-year follow-up than more-educated participants did. In contrast, we found no significant interaction of low compared to high education level in the intervention group, neither for changes in symptom severity from discharge to 3-month follow-up (B=0.21, SE 0.17, *P*=.07) nor for changes from discharge to 12-month follow-up (B=0.06, SE 0.15, *P*=.68). In contrast to the control group, less-educated intervention participants did not show a greater risk for deterioration in symptom severity than more-educated participants, indicating that participating in TIMT can effectively reduce this risk factor.

## Discussion

### Principal Results and Comparison With Prior Work

In the present study, we aimed to identify moderators of treatment outcome for TIMT following inpatient psychotherapy. Education level, positive outcome expectations, and mental health diagnoses were identified as significant moderators of TIMT’s effects on psychopathological symptom severity. Findings indicate that the effects of TIMT on general psychopathological symptom severity were more pronounced among participants with a low (vs high) education level. Participants with high positive outcome expectations profited in the short term (until 3-month follow-up) more than participants with low positive outcome expectations. However, this effect was not significant at 1-year follow-up. Moreover, participants with a mood disorder benefited less from the intervention than did participants with an anxiety disorder; however, this effect was also not significant at 1-year follow-up. Simple slope analyses revealed that even when some groups profited less from participating, treatment effects in these subgroups were still significant, except for the subgroup of participants with low positive outcome expectation at 3-month follow-up.

Other pretreatment variables did not interact with TIMT’s effects indicating that TIMT might be superior to TAU only with regard to outcome sustainability irrespective of age, gender, comorbid personality disorder, years since disorder onset, self-efficacy, remission status at the end of inpatient treatment, reliable change in psychopathological symptom severity during inpatient treatment, and Internet literacy. However, given that these analyses were exploratory and the study was not powered to find small interaction effects, these null findings should be interpreted with caution.

The finding that participants with low education benefited more from using TIMT than participants with high education contrasts with findings from a study investigating moderators in face-to-face continuation phase psychotherapy in which education did not interact with treatment outcome [27]. Moreover, the finding is also in contrast to 3 other studies, that found that high education was associated with a better treatment outcome in Internet-based intervention studies [23,29,50]. There are several possible explanations for the contrast between the current findings and findings from previous studies: First, these differences can be explained with variances in treatment type (acute vs maintenance phase; disorder-specific vs transdiagnostic), different type of acute phase treatment (outpatient vs inpatient), study population, and design. Second, it could also be hypothesized that inpatients with low education might display a higher risk for deterioration after inpatient discharge than those with high education because of their more pronounced difficulties with transferring the acquired skills into their daily life. Therefore, they might profit to a greater extent from a maintenance intervention than participants with high education. This assumption is in-line with a risk-reduction model of continuation phase treatments [8], assuming that such concepts may effectively reduce an increased risk for relapse or recurrence because of a nonchangeable vulnerability (eg, education, genetic predisposition, developmental conditions) by helping participants to reduce the consequences of such risk factors. Post hoc simple slope analyses revealed that in this study the control group of participants with low education were more likely to deteriorate compared to highly educated participants, whereas in the intervention group no such interaction could be found, indicating that participating in TIMT can effectively reduce this risk factor. Moreover, the inpatient treatments present the rather unique opportunity to introduce patients to the online-based intervention face-to-face and to teach them the necessary skills for using the intervention successfully. Therefore, 1 possible mechanism responsible for the findings in studies in which participants with low education profited less from Internet-based treatments (ie, low Internet skills) no longer has any effect. However, as this study is 1 of the first studies investigating moderators of outcomes in maintenance phase treatments following inpatient psychotherapy, future studies are clearly needed to further clarify the moderating role of education for treatment outcome.

On the basis of our data, we can only speculate on possible explanations as to why participants with anxiety disorder profited to a greater extent (in the short term) than participants with depression. These results are consistent with findings showing that effect sizes are typically larger for Internet interventions targeting anxiety than interventions targeting depression. In a review of 26 RCTs, Griffiths and colleagues [51] found that effect size differences ranged from 0.42 to 0.65 for interventions involving participants with clinically significant symptoms of depression, and 0.29 to 1.74 for interventions involving participants with a diagnosed anxiety disorder. Unlike guidelines for the treatment of depression [52,53], current guidelines for the treatment of anxiety disorders [1,54] do not recommend continuation phase psychological treatments following acute phase psychotherapy. Our findings, however, suggest that participants with anxiety disorder can benefit from an Internet-based maintenance treatment following inpatient psychotherapy. With regard to the subgroup effects for depression, future studies should try to examine treatment strategies to improve TIMT’s outcome, especially for this high-risk group [3]

The significant finding for positive outcome expectancies regarding change differences from discharge to 3-month follow-up is consistent with the idea that high expectancies for change are associated with better treatment outcome [45,55]. However, these change differences turned insignificant at 1-year follow-up. Therefore, its use as a predictive indicator for treatment allocation seems limited.

### Limitations

To validly interpret the results of this study, several limitations should be considered. First, as in most moderator studies, the analyses in this study were exploratory with participants not being randomized based on potential moderators of interest. Despite the limitations of this procedure, a growing recognition among methodologists has developed about its importance for fostering empirically founded hypotheses to be tested in future studies before clinical application [21]. Second, additional unmeasured variables (eg, participants’ genetic markers, developmental histories, self-regulation skills, coping strategies, attribution style, personality traits) may also moderate TIMT’s effects, which should be considered in subsequent studies. Third, as in most longitudinal studies, missing values had to be considered a relevant threat to the validity of the analyses. However, the adjustment used to address missing data (FIML) is especially robust with regard to missing data in mixed models [46]. Fourth, TIMT was a multicomponent intervention (ie, personal development plan, Web diary, peer support group, coach support, monitoring). Thus, the extent that the effects of specific components were moderated by studied variables is still unclear. Fifth, the sample size did not provide sufficient power to detect significant findings for potential moderator variables with subgroups of small sizes. Because of this limitation, other diagnoses in addition to mood disorder, adjustment, and anxiety disorders as potential moderating variables could not be included in the conducted analyses. Therefore, no generalization can be made for participants with other diagnoses. Likewise, the sample size did not provide sufficient power to examine moderators separately for different diagnostic status or gender. Therefore, it remains unclear whether moderators of TIMT’s effects vary across subpopulation (eg, different moderators for male and female or for different primary diagnoses). Compared with the nonparticipant cohort, individuals with low education were underrepresented in the study sample. Thus, the finding that TIMT was especially effective for participants with a low education level may only be generalizable to low-educated participants who are interested in participating in such an intervention. Finally, the sample included in this study was recruited in only 1 inpatient hospital, which clearly limits the generalizability of the findings to other patient populations.

### Strengths

Strengths of the study include (1) its large sample size compared to other studies, (2) a TAU control condition, which allowed us to specify which participants might and might not benefit from TIMT compared to treatment provided by routine health care services, (3) inclusion and exclusion criteria were kept to a minimum to maximize the ecological validity, and (4) generalizability of findings was assessed by comparing the moderator sample with a large sample of participants representing basically all patients treated in the study site.

### Conclusions

Transdiagnostic Internet-based guided self-help interventions may represent a cost-effective, far-reaching method for implementing maintenance phase treatments. Findings from the current study suggest that TIMT following inpatient psychotherapy helps patients differing in various characteristics to maintain treatment outcome. It is especially effective for participants with low education levels. Although some subgroups were identified as having profited less from the intervention than others, all subgroups benefited significantly. Future studies should replicate our results before clinical application.

## Multimedia Appendix 1

CONSORT-EHEALTH checklist V1.6.2 [56].

## Abbreviations

CBT: cognitive behavior therapy
FIML: full information maximum likelihood
ITT: intention-to-treat
RCT: randomized controlled trial
TAU: treatment as usual
TIMT: transdiagnostic Internet-based maintenance treatment
