# Supplementary material for: For Whom Does It Work? Moderators of Outcome on the Effect of a Transdiagnostic Internet-Based Maintenance Treatment After Inpatient Psychotherapy: Randomized Controlled Trial
Source: J Med Internet Res. 2013 Oct 10;15(10):e191. doi: 10.2196/jmir.2511 (PMC3849694; doi:10.2196/jmir.2511)
Supplement: Supplementary file 1 [file jmir_v15i10e191_app1.pdf]

# CONSORT-EHEALTH Checklist V1.6.2 Report

**(based on CONSORT-EHEALTH V1.6), available at [http://tinyurl.com/consort-ehealth-v1-6].**

2511

**Date completed**
12/23/2012 9:58:51
**by**
David Daniel Ebert

Is a Transdiagnostic Internet-based Maintenance Treatment after Inpatient Psychotherapy suitable for all Patients?

## TITLE

**1a-i) Identify the mode of delivery in the title**
Yes
"Is a Transdiagnostic Internet-based Maintenance Treatment after Inpatient Psychotherapy effective in all Patients? Moderators of Outcome from a Randomized Controlled Trial"

**1a-ii) Non-web-based components or important co-interventions in title**
Yes. "Is a Transdiagnostic Internet-based Maintenance Treatment after Inpatient Psychotherapy effective in all Patients? "
**1a-iii) Primary condition or target group in the title**
Yes.
"Is a Transdiagnostic Internet-based Maintenance Treatment after Inpatient Psychotherapy for Mental Health Disorders effective in all Patients? "

## ABSTRACT

**1b-i) Key features/functionalities/components of the intervention and comparator in the METHODS section of the ABSTRACT**
yes.

"TIMT involved an online self-management module, asynchronous patient-therapist communication, a peer support group, and an online-based progress monitoring. Participants in the TAU condition had unstructured access to outpatient psychotherapy, standardized outpatient face-to-face continuation treatment, and psychotropic management. "
**1b-ii) Level of human involvement in the METHODS section of the ABSTRACT**
"asynchronous patient-therapist communication,"
**1b-iii) Open vs. closed, web-based (self-assessment) vs. face-to-face assessments in the METHODS section of the ABSTRACT**
yes.
"The
study aimed to identify moderators of treatment outcome in a transdiagnostic internet-based maintenance treatment (TIMT) offered to patients after inpatient psychotherapy for mental disorders in routine care. "

"Self-reports of psychopathological symptoms and potential moderators were assessed at the start of inpatient treatment (t1), at discharge from inpatient treatment/start of TIMT (t2), and at 3-month (t3) and 12-month follow-up (t4)."
**1b-iv) RESULTS section in abstract must contain use data**
Only number of participants

"Using data from a randomized controlled trial (N = 400) designed to test the effectiveness of TIMT, we performed secondary analyses to identify factors moderating the effects of TIMT when compared with those of a treatment-as-usual control condition (TAU)."
**1b-v) CONCLUSIONS/DISCUSSION in abstract for negative trials**
not relevant, as there were no negative results

## INTRODUCTION

**2a-i) Problem and the type of system/solution**
"However, although there is evidence for the general effectiveness of Internet-based maintenance phase treatments, little is known about which patients might or might not benefit from this particular kind of treatment delivery. Investigating the moderating effects of patient characteristics on internet-based maintenance phase treatment effectiveness' is crucial for identifying appropriate populations and for customizing interventions to the specific needs of patient subgroups."

"The aim of the present study was to identify moderating factors on the effects of a transdiagnostic Internet-based maintenance treatment (TIMT) after inpatient psychotherapy for mental disorders. Using data from a pragmatic randomized controlled trial on the effectiveness of TIMT (ISRCTN:28632626) we conducted secondary analyses to identify demographic, clinical, and motivational variables that moderate the effects of TIMT on change in psychopathological symptom severity."
**2a-ii) Scientific background, rationale: What is known about the (type of) system**

Yes.
"Several studies have shown promising results with delivering maintenance phase treatments over the Internet (13-19) However, although there is evidence for the general effectiveness of Internet-based maintenance phase treatments, little is known about which patients might or might not benefit from this particular kind of treatment delivery"

"Using data from a pragmatic randomized controlled trial on the effectiveness of TIMT (ISRCTN:28632626) we conducted secondary analyses to identify demographic, clinical, and motivational variables that moderate the effects of TIMT on change in psychopathological symptom severity when compared with those of a treatment-as-usual control condition (TAU). "

## METHODS

**3a) CONSORT: Description of trial design (such as parallel, factorial) including allocation ratio**

Yes

"The aim of the present study was to identify moderating factors on the effects of a transdiagnostic Internet-based maintenance treatment (TIMT) after inpatient psychotherapy. "

"(1)Do any of the investigated pretreatment factors moderate the effectiveness of TIMT compared with TAU?
(2)If moderating effects are found, do participants characterized by disadvantageous scores on identified moderators still benefit from TIMT?"

**3b) CONSORT: Important changes to methods after trial commencement (such as eligibility criteria), with reasons**

there were no changes to methods after trial commencement

**3b-i) Bug fixes, Downtimes, Content Changes**

there were no bugifxes, downtimes or content changes during the trial

**4a) CONSORT: Eligibility criteria for participants**

"Patients were eligible for the study if they (a) were at least 18 years old, (b) met criteria for a mental disorder according to ICD-10 (c) spoke German sufficiently, (d) had basic reading and writing skills, and (e) had access to a computer with an Internet connection. Exclusion criteria were (a) a psychotic diagnosis, (b) acute alcohol or substance dependence, and (c) a significant risk for suicide. "

**4a-i) Computer / Internet literacy**

Computer / Internet literacy was not eligibility criteria, we also included patients with nor previous internet/computer experience.

**4a-ii) Open vs. closed, web-based vs. face-to-face assessments:**

" The RCT was conducted in a German clinic providing routine mental health care. "

"Design and results of the effectiveness trial are described in detail elsewhere (15)"

**4a-iii) Information giving during recruitment**

"Design and results of the effectiveness trial are described in detail elsewhere (15)"

**4b) CONSORT: Settings and locations where the data were collected**

" The RCT was conducted in a German inpatient clinic providing routine mental health care. "

**4b-i) Report if outcomes were (self-)assessed through online questionnaires**

"Study outcomes were assessed using self-report measures that were completed online at inpatient admission (t1), end of inpatient treatment/beginning of TIMT (t2), 3 months after discharge/end of TIMT (t3), and 12 months after inpatient treatment completion (t4)."

**4b-ii) Report how institutional affiliations are displayed**

no

**5) CONSORT: Describe the interventions for each group with sufficient details to allow replication, including how and when they were actually administered**

**5-i) Mention names, credential, affiliations of the developers, sponsors, and owners**

"The studied intervention was developed by the first author."

**5-ii) Describe the history/development process**

" In the effectiveness study, participants in the TIMT+TAU condition showed a better maintenance of inpatient treatment effects (i.e. differences in change of psychopathological symptom severity) from inpatient discharge to 3-month follow-up ($d = 0.38$, $P < .001$; CI: -0.35 ~ - 0.14) and 12-month follow-up ($d = 0.55$, $P < .001$; CI: -0.50 ~ - 0.22) than TAU-only controls. Design and results of the effectiveness trial are described in detail elsewhere (Ebert et al., in press)."

**5-iii) Revisions and updating**

The studied intervention is the first version. There are no other version. There were no major changes during the evaluation process. Content was frozen during the trial.

**5-iv) Quality assurance methods**

"Coaches were supervised once a week by a licensed senior therapist, as is usual in the study hospital"

**5-v) Ensure replicability by publishing the source code, and/or providing screenshots/screen-capture video, and/or providing flowcharts of the algorithms used**

"Design and results of the effectiveness trial are described in detail elsewhere (15)"

**5-vi) Digital preservation**

"Design and results of the effectiveness trial are described in detail elsewhere (15)"

**5-vii) Access**

"The RCT was conducted in a German clinic providing routine mental health care. "
"Design and results of the effectiveness trial are described in detail elsewhere (15)"

**5-viii) Mode of delivery, features/functionalities/components of the intervention and comparator, and the theoretical framework**

"Interventions
Inpatient Treatment.
Inpatient treatment was based on Cognitive Behavioral Therapy [22]. Patients received one session of individual therapy (50 minutes) and an average of six sessions of group therapy (a 90 minutes) per week. Interventions were supplemented with sports- and physiotherapy, as well as medical treatment (including pharmacotherapy) when necessary. Treatment was delivered by six experienced therapists and 14 therapists in training. Duration of treatment ranged between 22 and 98 days (M = 46.30, SD = 8.17).
TAU-only Condition.
Following inpatient treatment, all participants had unstructured access to outpatient psychotherapy and standardized outpatient group-based face-to-face maintenance treatment 23, as typically provided by the referring agencies. In addition, there was no restriction on the use of medication during the study period.
TIMT+TAU Condition.
In addition to TAU, the TIMT+TAU group had a 12-week transdiagnostic Internet-based maintenance treatment (TIMT). The main focus of TIMT is to support patients in the sustained utilization of skills acquired during treatment. For this purpose, TIMT works to help patients to identify activities which they have found helpful and systematically integrate these into their daily-life routines. Since TIMT aims to enhance whatever strategy patients experienced as helpful, it can be used to maintain treatment outcome regardless of which psychopathology the patient is suffering from and regardless of the kind of treatment the patient received before. TIMT consists of five core components. The first component is the generation of a Personal Development Plan. This process is conducted during the last ten days of inpatient treatment, in which TIMT participants complete three sessions of blended (face-to-face and online) standardized goal-setting and action planning instead of inpatient treatment as usual. Participants develop a detailed plan including (a) highly relevant personal goals they want to achieve during the intervention phase, and (b) implementation intentions [24], including details on how and when they will achieve these goals. The second and central component of TIMT is the completion of a structured Web Diary in which participants weekly evaluate the realization of their personal goals and set specific goals for the next week. The third component of TIMT is an online Peer-Support Group. Subgroups consisting of 3 to 6 participants are asked to give asynchronous online feedback to each other on their web diaries. The fourth component of TIMT is Coach Support, involving weekly asynchronous written online feedback from a therapist regarding a patients' web diary. Finally, TIMT includes weekly online Monitoring of psychopathological symptoms."

**5-ix) Describe use parameters**

"The second and central component of TIMT is the completion of a structured Web Diary in which participants weekly evaluate the realization of their personal goals and set specific goals for the next week."

**5-x) Clarify the level of human involvement**

"The fourth component of TIMT is Coach Support, involving weekly asynchronous written online feedback from a therapist regarding a patients' web diary. Coaches were advised not to spend more than 30 minutes per week on support per patient. Total duration of support rendered was 231 minutes
on average per patient (10-490; SD = 128)."

**5-xi) Report any prompts/reminders used**

coaches reminded participants on a weekly, in case they didn´t used the intervention in the last week.

**5-xii) Describe any co-interventions (incl. training/support)**

"Inpatient Treatment. Inpatient treatment was generally based on Cognitive Behavioral Therapy [27]. Patients received one session of individual therapy (50 minutes) and an average of six sessions of group therapy (90 minutes) per week. Interventions were supplemented with sports- and physiotherapy, as well as medical treatment (including medication) when necessary. Treatment was delivered by six experienced therapists and 14 therapists in training. Duration of treatment ranged between 22 and 98 days (M = 46.30, SD = 8.17).
TAU-only Condition. Following inpatient treatment, all participants had unstructured access to outpatient psychotherapy and standardized outpatient group-based face-to-face maintenance treatment [8], typically provided by the referring agencies. In addition, there was no restriction on the use of medication during the study period. "

**6a) CONSORT: Completely defined pre-specified primary and secondary outcome measures, including how and when they were assessed**

"Dependent variable.
The primary outcome from the effectiveness trial was change in general psychopathological symptom severity (PSS) from discharge (t2) to a 3- and 12-month follow-up (t3, t4). PSS was assessed using the HEALTH-49, a widely used measure of symptom severity in Germany 26. The Health-49 PSS scale consists of 18 items related to somatoform complaints (7 items), depressiveness (6 items), and phobic anxiety (5 items). Participants were asked to rate the severity in which they had suffered from the presented symptoms in the previous two weeks (0 = not at all, 4 = very much). Reliability and construct validity have been established in several studies based on large clinical and non-clinical samples (n = 1548 psychotherapy inpatients, n = 5630 primary-care patients, see (26). In the present study, internal consistency (Cronbach's coefficients) at baseline was .87 for the overall score (PSS), 0.90 for depressive symptoms, 0.86 for somatoform complaints, and 0.86 for phobic anxiety."

"Study outcomes were assessed using self-report measures that were completed at inpatient admission (t1), end of inpatient treatment/beginning of TIMT (t2), 3 months after discharge/end of TIMT (t3), and 12 months after inpatient treatment completion (t4)."

**6a-i) Online questionnaires: describe if they were validated for online use and apply CHERRIES items to describe how the questionnaires were designed/deployed**

no

**6a-ii) Describe whether and how "use" (including intensity of use/dosage) was defined/measured/monitored**

"(completed at least 6 of 12 web diary entries or more than 25 posts, n = 177)."

""Design and results of the effectiveness trial are described in detail elsewhere (15)"

**6a-iii) Describe whether, how, and when qualitative feedback from participants was obtained**

Qulitative Feedback was obtained, but not reported here in this study (as it is not important for the research question (moderators) of this study.

**6b) CONSORT: Any changes to trial outcomes after the trial commenced, with reasons**

there were no changes to the trial outcomes.

**7a) CONSORT: How sample size was determined**

**7a-i) Describe whether and how expected attrition was taken into account when calculating the sample size**

"The study was powered to find a small to moderate effect size in the main effect analyses which was considered to be the smallest relevant difference to health-care decision makers in this context. "

ITT was applied, therefore there was no need to take attrition into account.

**7b) CONSORT: When applicable, explanation of any interim analyses and stopping guidelines**

there were no stopping guidelines or interim analyses.

**8a) CONSORT: Method used to generate the random allocation sequence**

""Design and results of the effectiveness trial are described in detail elsewhere (15)"

Randomization was performed by blindly drawing a random sample from shuffled consent forms that patients had completed after the group-based introductory session. The selection of forms was conducted by blinded administrative staff not otherwise involved in the study. Results of the randomization process were first revealed to patients and therapists no earlier than three days before treatment started.

**8b) CONSORT: Type of randomisation; details of any restriction (such as blocking and block size)**

no restrictions applied.

**9) CONSORT: Mechanism used to implement the random allocation sequence (such as sequentially numbered containers), describing any steps taken to conceal the sequence until interventions were assigned**

Randomization was performed by blindly drawing a random sample from shuffled consent forms that patients had completed after the group-based introductory session. The selection of forms was conducted by blinded administrative staff not otherwise involved in the study. Results of the randomization process were first revealed to patients and therapists no earlier than three days before treatment started.

**10) CONSORT: Who generated the random allocation sequence, who enrolled participants, and who assigned participants to interventions**

Randomization was performed by blindly drawing a random sample from shuffled consent forms that patients had completed after the group-based introductory session. The selection of forms was conducted by blinded administrative staff not otherwise involved in the study. Results of the randomization process were first revealed to patients and therapists no earlier than three days before treatment started.

**11a) CONSORT: Blinding - If done, who was blinded after assignment to interventions (for example, participants, care providers, those assessing outcomes) and how**

**11a-i) Specify who was blinded, and who wasn't**

outcomes were assessed using self--report measures. Therefore blinding was not needed. Participants and Coaches were not blind to treatment conditions.

**11a-ii) Discuss e.g., whether participants knew which intervention was the "intervention of interest" and which one was the "comparator"**

Participants and Coaches were not blind to treatment conditions.

**11b) CONSORT: If relevant, description of the similarity of interventions**

not relevant, as control condition was treatment as usual.

**12a) CONSORT: Statistical methods used to compare groups for primary and secondary outcomes**

Interactions between pretreatment patient characteristics (moderators) and interindividual differences in intraindividual changes across measurement occasions were modeled and tested via multilevel mixed effect models (MEM). Change in PSS over time was dummy-coded and treated as a fixed level-1 (i.e., within-subjects) effect (Dummy1: T1-T2, Dummy2: T2-T3, Dummy3: T2-T4). Treatment conditions (0 = control condition, 1 = intervention condition) was treated as a fixed level-2 (i.e., between-subjects) effect. More important for the present purpose, interactions between moderator and treatment condition, all cross-level interaction effects "(condition x T1-T2 / T2-T3 / T2-T4, moderator x T1-T2 / T2-T3 / T2-T4), and three-way interaction effects (moderator x condition x T1-T2 / T2-T3 / T2-T4) were also included in the models. A three-way interaction effect of moderator x condition x T2-T3 or moderator x condition x T2-T4 would indicate that the magnitude of the intervention effect varies as a function of the moderator. The model imposed no restrictions on the covariance matrix for measurement occasions. Thus, no model assumptions were tested. We standardized continuous predictors so that regression coefficients were estimated for participants with average scores on the putative moderator. "

**12a-i) Imputation techniques to deal with attrition / missing values**

"We employed a full information maximum likelihood estimation (FIML), which allows for all available data to be included without replacement or imputation of missing values. FIML estimation for mixed models is especially robust with respect to missing data (29)"

**12b) CONSORT: Methods for additional analyses, such as subgroup analyses and adjusted analyses**

"Additionally, we conducted follow-up simple slope analyses for each significant three-way interaction effect (30) to probe the relevant lower-order effects. In this method, the slope and the significance of the intervention main effect is evaluated for conditional values of the moderator. For significant three-way interactions of continuous moderators, simple slopes were calculated for the mean and one standard deviation above and below the mean (31). "

"To verify whether the results of the ITT analyses would be sustained among the intervention completers sample only, we subsequently repeated all MEM with participants who stayed within key treatment parameters (completed at least 6 of 12 web diary entries or more than 25 posts, n = 177)."

"Finally, if a significant moderator effect contradicted our a priori expectations, we conducted post-hoc simple slope analyses for the TAU group and the intervention group separately in order to identify the reasons for the effect. "

## RESULTS

**13a) CONSORT: For each group, the numbers of participants who were randomly assigned, received intended treatment, and were analysed for the primary outcome**

Figure 1: TIMT+TAU: "Analysed ITT: n=200, Excluded from analysis (n=0)"
TAU: "Analysed ITT: n=200, Excluded from analysis (n=0)"

**13b) CONSORT: For each group, losses and exclusions after randomisation, together with reasons**

Figure 1: TIMT+TAU
"Lost to follow-up at 3 months (n=28; 14%)

-Discontinued intervention & no data provided at follow up (n=7; 3.5%)
-Completed intervention & no data provided at follow-up (n=21; 10.5%)"

TAU-only: "Lost to follow-up at 3 months (n=30; 15%)

-No data provided at follow-up (n=30, 15%)"

TIMT+TAU: "Lost to follow-up at 12 months (n=69; 34.5%)

-Discontinued intervention & no data provided at follow up (n=12; 6%)
-Completed intervention & no data provided at follow-up (n=57; 28.5%)"

TAU-only: "Lost to follow-up at 12 months (n=54; 22%)

- No data provided (n=54; 22%)"

**13b-i) Attrition diagram**

"Discontinued intervention (n=31; 15.5%)

-Technical problems/no internet (n=6; 3%)
-Lack of time (n=4; 2%)
-Lack of energy (n=3; 1,5%)
-Questioned usefulness (n=2; 1%)
-Change to f2f-therapy only (n=2; 1%)
-No information about reasons (n=14; 7%) "

**14a) CONSORT: Dates defining the periods of recruitment and follow-up**

"We recruited potential participants from 2189 patients treated for a variety of mental disorders between July 2008 and October 2009 in the study hospital."

**14a-i) Indicate if critical “secular events” fell into the study period**

6 participants in the intervention reported that they could not use the intervention as they had lost their internet connection.

Figure 1: "-Technical problems/no internet (n=6; 3%)"

**14b) CONSORT: Why the trial ended or was stopped (early)**

trial was not stopped early.

**15) CONSORT: A table showing baseline demographic and clinical characteristics for each group**

yes. Table 2 in the manuscript.

"Age (mean, SD)
Sex (female, %)
Education (%), high, medium, low
Existing internet literacy (%)
Disorder (%), Depression, Anxiety, Adjustment, Other
Comorbid PD (%)
Years since first disorder onset (years, %), < 1 year, 1-5 years, > 5 years
Reliable change during inpatient treatment (%)
Remission at discharge (%)
Self-efficacy (mean, SD)
Positive outcome expectations (mean, SD)"

**15-i) Report demographics associated with digital divide issues**

reported in Table 2:

"Age (mean, SD)
Sex (female, %)
Education (%), high, medium, low
Existing internet literacy (%)"

**16a) CONSORT: For each group, number of participants (denominator) included in each analysis and whether the analysis was by original assigned groups**
**16-i) Report multiple "denominators" and provide definitions**
"To verify whether the results of the ITT analyses would be sustained among the intervention completers sample only, we subsequently repeated all MEM with participants who stayed within key treatment parameters (completed at least 6 of 12 web diary entries or more than 25 posts, n = 177)."

"Intervention Completers Sample
The results of the following intervention completers analyses closely paralleled those of the ITT analyses. Most of the significant three-way interactions were also significant in the completers sample (B = -0.45 – -0.12, SE = 0.05 – 0.21, P = 0.031 – 0.046). Only the interaction of education dummy 2 with condition  T2-T4 was no longer significant at follow-up (B = -0.43, SE = 0.22, P = 0.053). None of the non-significant interactions in the ITT analyses was significant in the completers sample (B = -0.11 – 0.02, SE = 0.07 – 0.20, P = 0.078 – 0.97). "

"Effect Sizes.
Effect sizes (ES, Cohen's d) for each significant moderator were calculated based on comparing the effect of control versus intervention condition on PSS, with participants grouped by parameter values on each significant moderator variable. A mean ES of d = 0.22 was found for patients with high education and d = 0.80 for patients with low education for TAU versus TIMT+TAU group differences in change of psychopathological symptom severity from discharge to 3-month follow up. For change from discharge to 1-year follow-up, a mean ES of d = 0.30 for high educated patients and a mean ES of d = 0.57 for low educated patients were found. With diagnoses as the moderator, control-versus-intervention group differences in change from discharge to 3-month follow-up were d = 0.33 for patients with a mood disorder and d = 1.02 for patients with an anxiety disorder. With positive outcome expectations as moderator, control versus intervention group differences in change from discharge to 3-month follow-up were d = 0.58 for patients with high positive outcome expectations, d = 0.39 for patients with mean positive outcome expectations, and d = 0.20 for patients with low positive outcome expectations. "

**16-ii) Primary analysis should be intent-to-treat**
"Aiming at an intention-to-treat design, we included all participants randomly assigned to conditions. "

"To verify whether the results of the ITT analyses would be sustained among the intervention completers sample only, we subsequently repeated all MEM with participants who stayed within key treatment parameters (completed at least 6 of 12 web diary entries or more than 25 posts, n = 177)."
**17a) CONSORT: For each primary and secondary outcome, results for each group, and the estimated effect size and its precision (such as 95% confidence interval)**
We report always the regression coefficient and additionally the standard error.
**17a-i) Presentation of process outcomes such as metrics of use and intensity of use**
"Design and results of the effectiveness trial are described in detail elsewhere (15)"

"(completed at least 6 of 12 web diary entries or more than 25 posts, n = 177)."

Reporting process outcome would go beyond the scope of the paper, as the scope is to investigate pretreatment patient characteristics as moderators of treatment outcome.
**17b) CONSORT: For binary outcomes, presentation of both absolute and relative effect sizes is recommended**
There were no binary outcomes in this study.
**18) CONSORT: Results of any other analyses performed, including subgroup analyses and adjusted analyses, distinguishing pre-specified from exploratory**
Post-hoc analyses demonstrated that although simple slopes for the intervention main effects (condition  T2-T3 / T2-T4) were lower among high educated patients compared to low educated patients, the intervention main effect was still significant (simple slope high educated patients T2-T3: B = -.17, SE B = 08, P = .041, T2-T4: B = -.25, SE B = 11, P = .026, simple slope low educated patients T2-T3: B = -.49, SE B = 14, P < 0.001, T2-T4: B = -.66, SE B = 18, P < .001). "

"Post-hoc analyses demonstrate that although simple slopes for the intervention main effect (condition  T2-T3) were lower among patients with a mood disorder compared to patients with an anxiety disorder, the intervention main effect was significant in both groups (simple slope mood disorder T2-T3: B = -.21, SE B = .07, P = .004, simple slope anxiety disorder T2-T3: B = -.64, SE B = .02, P < 0.001)."

"). Follow-up analyses revealed that although simple slopes for the intervention main effect (condition  T2-T3) were lower among patients with a moderate (mean) positive outcome expectation than for patients with a high (mean +1 SD) positive outcome expectations, the intervention effect was still significant (simple slope moderate positive outcome expectations T2-T3: B = -.25, SE B = .05, P < .001, simple slope high positive outcome expectations T2-T3: B = -.36, SE B = .07, P < 0.001). For patients with a low positive outcome expectations (mean -1 SD) the simple slope for the intervention main effect (condition  T2-T3) was lower and no longer significant (simple slope low positive outcome expectations [mean -1 SD] T2-T3: B = -.13, SE B = .07, P < 0.093). Only 14.4% of patients (N = 57) expressed low positive outcome expectations. Therefore, the drop to non-significance was likely due to low power. Moreover, simple slope analyses for this patient group showed that the intervention main effect on change in PSS from discharge to 1-year follow-up was highly significant (simple slope low positive outcome expectations [mean -1 SD] T2-T4: B = -.38, SE B = .10, P < 0.001). "

"Post-hoc-analyses
The moderator effect of education contradicted our a priori expectation of higher educated patients benefiting to a greater extent from the internet-based intervention than lower educated patients. Thus, we conducted further post-hoc simple slope analyses for the TAU group and the intervention group separately to identify possible explanations for this effect. For patients in the TAU-only group, we found no significant interaction between education and changes in PSS from discharge to 3-month follow-up (education dummy 2  T2-T3 interaction, B = .10, SE B = .11, P = .351), but we found a significant interaction between education and changes from discharge to 12-month follow-up (education dummy 2  T2-T4, B = .35, SE B = .14, P = .015). Less educated patients had a greater risk for deterioration from discharge to 1-year follow-up than more educated patients. In contrast, we found no significant interaction of low compared to high education level in the TIMT + TAU group, neither for changes in PSS from discharge to 3-month follow-up (B = .21, SE B = .17, P = .07) nor for changes from discharge to 12-month follow-up (B = .06, SE B = .15, P = .68). In contrast to the TAU group, less educated TIMT + TAU participants did not show a greater risk for deterioration in PSS than more educated participants, indicating that participating in TIMT can effectively reduce this risk factor."

**18-i) Subgroup analysis of comparing only users**
Intervention Completers Sample
"The results of the following intervention completers analyses closely paralleled those of the ITT analyses. Most of the significant three-way interactions were also significant in the completers sample (B = -0.45 – -0.12, SE = 0.05 – 0.21, P = 0.031 – 0.046). Only the interaction of education dummy 2 with condition T2-T4 was no longer significant at follow-up (B = -0.43, SE = 0.22, P = 0.053). None of the non-significant interactions in the ITT analyses was significant in the completers sample (B = -0.11 – 0.02, SE = 0.07 – 0.20, P = 0.078 – 0.97). "

**19) CONSORT: All important harms or unintended effects in each group**
No adverse events were reported.
**19-i) Include privacy breaches, technical problems**
6 participants in the intervention reported that they could not use the intervention as they had lost their internet connection.

Figure 1: "-Technical problems/no internet (n=6; 3%)"
**19-ii) Include qualitative feedback from participants or observations from staff/researchers**
Reporting qualitative feedback from participants would go beyond the scope of the paper (as it is abourt identiffying pretreatment factors as moderator of the effectiveness)

## DISCUSSION

**20) CONSORT: Trial limitations, addressing sources of potential bias, imprecision, multiplicity of analyses**
**20-i) Typical limitations in ehealth trials**
"To validly interpret the results of this study, several limitations should be considered. First, as in most moderator studies, the analyses in this study were exploratory with patients not being randomized based on potential moderators of interest. Despite the limitations of this procedure, a growing recognition among methodologists has developed about its importance for fostering empirically founded hypotheses to be tested in future studies before clinical application 20. Second, additional unmeasured variables (e.g., patients' genetic markers, developmental histories, self-regulation skills, coping strategies, attribution style, personality traits) may also moderate TIMT's effects, which should be considered in subsequent studies. Third, as in most longitudinal studies, missing values had to be considered a relevant threat to the validity of the analyses. However, the adjustment used to address missing data (FIML) is especially robust with regard to missing data in mixed models 29. Fourth, the sample size did not provide sufficient power to detect significant findings for potential moderator variables with subgroups of small sizes. Because of this limitation, other diagnoses besides mood disorder, adjustment, and anxiety disorders as potential moderating variables could not be included in the conducted analyses. Therefore, no generalization can be made for patients with other diagnoses. Likewise the sample size did not provide sufficient power to examine moderators seperately for different diagnostic status or gender. Therefore, it remains unclear whether moderators of TIMT's effects vary across subpopulation (e.g., different moderators for male and female or for different primary diagnoses). "
**21) CONSORT: Generalisability (external validity, applicability) of the trial findings**
**21-i) Generalizability to other populations**
"(d) generalizability of findings was assessed by comparing the moderator sample with a large sample of patients representing basically all patients treated in the study site. "
**21-ii) Discuss if there were elements in the RCT that would be different in a routine application setting**
The study is based on a pragmatic randomized controlled trial, implemented in routine care of an inpatient hospital in Germany and conducted under routine care conditions.
**22) CONSORT: Interpretation consistent with results, balancing benefits and harms, and considering other relevant evidence**
**22-i) Restate study questions and summarize the answers suggested by the data, starting with primary outcomes and process outcomes (use)**
"In the present study we aimed to identify moderators of treatment outcome in a transdiagnostic internet-based maintenance treatment following inpatient psychotherapy.
Education level, positive outcome expectations, and mental health diagnoses were identified as significant moderators of TIMT's effects on psychopathological symptom severity. Findings indicate that the effects of TIMT on PSS were more pronounced among participants with a low (compared to high) education level. Patients with high positive outcome expectations profited in the short term (until 3-month follow-up) more than patients with low positive outcome expectations. However, this effect was not significant at 1-year follow-up. Moreover, patients with a mood disorder benefitted less from the intervention than did patients with an anxiety disorder; however, this effect was also not significant at 1-year follow-up. Simple slope analyses revealed that even when some groups profited less from participating, treatment effects in these subgroups were still significant, except for the subgroup of patients with low positive outcome expectation at 3-month follow-up.
Other pretreatment variables did not interact with TIMT's effects indicating that TIMT is superior to TAU-only with regard to outcome sustainability irrespective of age, gender, comorbid personality disorder, years since first disorder onset, self-efficacy, remission status at the end of inpatient treatment, reliable change in psychopathological symptom severity during inpatient treatment, and internet literacy."
**22-ii) Highlight unanswered new questions, suggest future research**
"Because of this limitation, other diagnoses besides mood disorder, adjustment, and anxiety disorders as potential moderating variables could not be included in the conducted analyses. Therefore, no generalization can be made for patients with other diagnoses. Likewise the sample size did not provide sufficient power to examine moderators seperately for different diagnostic status or gender. Therefore, it remains unclear whether moderators of TIMT's effects vary across subpopulation (e.g., different moderators for male and female or for different primary diagnoses). "

## Other information

**23) CONSORT: Registration number and name of trial registry**
"Trial Registration: International Standard Randomized Controlled Trial Number (ISRCTN): 28632626"
**24) CONSORT: Where the full trial protocol can be accessed, if available**
not available.
**25) CONSORT: Sources of funding and other support (such as supply of drugs), role of funders**
"This study was funded by the Dr. Ebel Fachkliniken – Vogelsbergklinik, Grebenhain, Germany and the European Union (EFRE: CCI 2007DE161PR001)"

Funders were not involved in data-analyses or interpretation.
**X26-i) Comment on ethics committee approval**
"All procedures were approved by the university and the hospital institutional review boards"
**x26-ii) Outline informed consent procedures**
Informed consent was obtained offline.
**X26-iii) Safety and security procedures**

To account for potential suicidal crises in this high risk group, we offered a 24-hour emergency telephone hotline (which was unexpectedly not used by a single patient). Access to the platform proceeded through a unique username password combination and was available 24/7. All transferred data were secured via AES-256 encryption.

**X27-i) State the relation of the study team towards the system being evaluated**

"The first author developed the intervention under study."
